# Supplementary material for: Digital proximity tracing on empirical contact networks for pandemic control
Source: Nat Commun. 2021 Mar 12;12:1655. doi: 10.1038/s41467-021-21809-w (PMC7955065; doi:10.1038/s41467-021-21809-w)
Supplement: Supplementary file 2 — Reporting Summary [file 41467_2021_21809_MOESM2_ESM.pdf]

nature research

Corresponding author(s): Bruno Lepri

Last updated by author(s): Feb 3, 2021

Reporting Summary

Nature Research wishes to improve the reproducibility of the work that we publish. This form provides structure for consistency and transparency in reporting. For further information on Nature Research policies, see our Editorial Policies and the Editorial Policy Checklist.

Statistics

For all statistical analyses, confirm that the following items are present in the figure legend, table legend, main text, or Methods section.

n/a	Confirmed	
	x	The exact sample size (n) for each experimental group/condition, given as a discrete number and unit of measurement
	x	A statement on whether measurements were taken from distinct samples or whether the same sample was measured repeatedly
x		The statistical test(s) used AND whether they are one- or two-sided<br>*Only common tests should be described solely by name; describe more complex techniques in the Methods section.*
x		A description of all covariates tested
	x	A description of any assumptions or corrections, such as tests of normality and adjustment for multiple comparisons
	x	A full description of the statistical parameters including central tendency (e.g. means) or other basic estimates (e.g. regression coefficient) AND variation (e.g. standard deviation) or associated estimates of uncertainty (e.g. confidence intervals)
x		For null hypothesis testing, the test statistic (e.g. F, t, r) with confidence intervals, effect sizes, degrees of freedom and P value noted<br>*Give P values as exact values whenever suitable.*
x		For Bayesian analysis, information on the choice of priors and Markov chain Monte Carlo settings
x		For hierarchical and complex designs, identification of the appropriate level for tests and full reporting of outcomes
x		Estimates of effect sizes (e.g. Cohen's d, Pearson's r), indicating how they were calculated

Our web collection on statistics for biologists contains articles on many of the points above.

Software and code

Policy information about availability of computer code

Data collection	We have not collected data for our study but we have used data publicly available
Data analysis	The custom code was developed in Python. In particular, we have used numpy, scipy, networkx, and matplotlib. Custom code is made available on GitHub at the following link: https://github.com/DigitalContactTracing/covid_code

For manuscripts utilizing custom algorithms or software that are central to the research but not yet described in published literature, software must be made available to editors and reviewers. We strongly encourage code deposition in a community repository (e.g. GitHub). See the Nature Research guidelines for submitting code & software for further information.

Data

Policy information about availability of data

All manuscripts must include a data availability statement. This statement should provide the following information, where applicable:
- Accession codes, unique identifiers, or web links for publicly available datasets
- A list of figures that have associated raw data
- A description of any restrictions on data availability

We are pleased to make available the source-code and datasets accompanying this research.
The code is available at https://github.com/DigitalContactTracing/covid_code,
the CNS data (already publicly available) at https://doi.org/10.6084/m9.figshare.7267433
and the SocioPatterns data (already publicly available) at http://www.sociopatterns.org

Field-specific reporting

Please select the one below that is the best fit for your research. If you are not sure, read the appropriate sections before making your selection.

☐ Life sciences ☒ Behavioural & social sciences ☐ Ecological, evolutionary & environmental sciences

For a reference copy of the document with all sections, see nature.com/documents/nr-reporting-summary-flat.pdf

Behavioural & social sciences study design

All studies must disclose on these points even when the disclosure is negative.

Study description	We have developed and evaluated an epidemic spreading and tracing model based on quantitative data of real social contacts.
Research sample	In our experiments we have used publicly available data. More specifically, we have used the Copenhagen Network Study (CNS) dataset and two SocioPatterns datasets. The CNS dataset collect proximity data among university students and can be accessed at the following link: https://doi.org/10.6084/m9.figshare.7267433<br>The two SocioPatterns datasets are collected in a high school and in a workplace. Data can be accessed at the following link: http://www.sociopatterns.org
Sampling strategy	We have used the state of the art available datasets. We focused most of the analyses on the most complete one, the CNS dataset, representing a university campus. We then reported results for two different environments too: a High school and a workplace, in order to generalize our conclusions.
Data collection	The CNS dataset was collected as part of a large observational project, where the network data set, which also includes many other modalities in addition to Bluetooth signal strengh (phone calls, text messages, Facebook friendships, etc), was collected via smartphones across multiple years (2013-2016).<br>See https://journals.plos.org/plosone/article?id=10.1371/journal.pone.0095978 and https://www.nature.com/articles/s41597-019-0325-x for full details on the dataset.<br>Also note that the data collection was performed with explicit consent from all participants and approved by the Danish Data Protection Agency, which is the relevant legal entity in Denmark.<br><br>The SocioPatterns datasets are collected as part of a decade-long research collaboration that uses wearable proximity sensors to measure time-resolved human proximity networks in a variety of real-world environments relevant for airborne disease transmission, including hospitals (https://journals.plos.org/plosone/article?id=10.1371/journal.pone.0017144), primary schools (https://journals.plos.org/plosone/article?id=10.1371/journal.pone.0023176), high schools (https://journals.plos.org/plosone/article?id=10.1371/journal.pone.0136497), large social gatherings (https://www.sciencedirect.com/science/article/abs/pii/S0022519310006284), households (https://journals.plos.org/plosone/article?id=10.1371/journal.pone.0198733), and more. Several of these datasets have been made available to the public (http://www.sociopatterns.org/datasets/).<br>Specifically, the datasets used here were collected and made available to the public in studies reported below:<br>* "high school" SocioPatterns dataset<br>- paper: https://journals.plos.org/plosone/article?id=10.1371/journal.pone.0136497<br>- public dataset: https://doi.org/10.1371/journal.pone.0136497.s002<br>- 327 students consented to participate in the study (out of 379, a 86.3% participation rate) during the week of Dec. 2-6, 2013<br>- informed consent was obtained and recorded from all participants in the study.<br>* offices ("INVS15") SocioPatterns dataset:<br>- paper: https://epjdatascience.springeropen.com/articles/10.1140/epjds/s13688-018-0140-1<br>- public dataset: http://www.sociopatterns.org/datasets/co-location-data-for-several-sociopatterns-data-sets/<br>- 211 individuals consented to participate in the study, over about 12 days in 2015<br>- informed consent was obtained and recorded from all participants in the study.<br><br>The research reported in the present study occurred subsequent to data collection, and consisted of epidemic simulations on the empirical time-resolved proximity network. The part of our study pertaining to the CNS and the SocioPatterns network dataset did not have "experimental conditions", nor explicitly stated hypotheses.
Timing	The CNS dataset is described in full detail in https://www.nature.com/articles/s41597-019-0325-x. In that publication, we argue that we cannot reveal the exact start and stop date of the data collection period since it would make it easier to re-identify individuals in the data set. We can, however, reveal that the data was collected over a month in early 2014.<br>The Sociopatterns High School dataset was collected in December 2013 and the office one from June 24 to July 3, 2013.
Data exclusions	As mentioned above, detailed data processing for CNS is discussed in detail here: https://www.nature.com/articles/s41597-019-0325-x. Some students with very little recorded data (e.g. due to broken smartphone hardware or) were excluded from the dataset in order to ensure high coverage for each individual in the data set.<br>Further, the timing of Bluetooth scans (which in the raw data have second-level time-stamp) was quanitzed into 5 minute bins, and the bluetooth signal strength was symmetrized: within each time bin, we found all instances of users A and B discovering each other, reported the one with the highest RSSI, and discarded others; and the information of directionality (whether user A discovered user B or vice versa) was discarded.
Non-participation	As mentioned above, in the CNS dataset we have excluded data from users who had very low data quality. This matter is discussed in

	detail https://www.nature.com/articles/s41597-019-0325-x (Fig 2a shows the distribution of missing data per user). In the study presented here, we keep 706 out of a total of 850 users with recorded activity.
Randomization	Participants were not allocated into experimental groups.

Reporting for specific materials, systems and methods

We require information from authors about some types of materials, experimental systems and methods used in many studies. Here, indicate whether each material, system or method listed is relevant to your study. If you are not sure if a list item applies to your research, read the appropriate section before selecting a response.

Materials & experimental systems

n/a	Involved in the study
☒	☐ Antibodies
☒	☐ Eukaryotic cell lines
☒	☐ Palaeontology and archaeology
☒	☐ Animals and other organisms
☒	☐ Human research participants
☒	☐ Clinical data
☒	☐ Dual use research of concern

Methods

n/a	Involved in the study
☒	☐ ChIP-seq
☒	☐ Flow cytometry
☒	☐ MRI-based neuroimaging
